# High pneumococcal density correlates with more mucosal inflammation and reduced respiratory syncytial virus disease severity in infants

**DOI:** 10.1186/s12879-016-1454-x

**Published:** 2016-03-17

**Authors:** Marloes Vissers, Inge M. Ahout, Corné H. van den Kieboom, Christa E. van der Gaast de Jongh, Laszlo Groh, Amelieke J. Cremers, Ronald de Groot, Marien I. de Jonge, Gerben Ferwerda

**Affiliations:** Laboratory of Pediatric Infectious Diseases, Department of Pediatrics, Radboud Institute for Molecular Life Sciences, Radboud University Medical Center, P. O. Box 9101, 6500 HB Nijmegen, The Netherlands

**Keywords:** Respiratory syncytial virus, *Streptococcus pneumoniae*, Disease severity, Nasopharyngeal colonization, MMP-9

## Abstract

**Background:**

Respiratory syncytial virus (RSV) is an important cause of lower respiratory tract infections in infants. A small percentage of the infected infants develops a severe infection, while most of these severely ill patients were previously healthy. It remains unclear why these children develop severe RSV infections. In this study, we investigate whether pneumococcal nasopharyngeal carriage patterns correlate with mucosal inflammation and severity of disease.

**Methods:**

In total, 105 infants hospitalized with RSV infection were included and recovery samples were taken from 42 patients. The presence and density of *Streptococcus pneumoniae* was determined by RT qPCR to study its relation to viral load, inflammation (MMP-9 and IL-6) and severity of RSV disease.

**Results:**

We show that pneumococcal presence or absence in the nasopharynx does not correlate with viral load, inflammation or severity of disease. However, when pneumococcus is present in patients, a higher nasopharyngeal pneumococcal density was correlated with a higher RSV load, higher MMP-9 levels and a less severe course of disease.

**Conclusions:**

Our results show correlations between *S. pneumoniae* density and viral load, inflammation and disease severity, suggesting that pneumococcal density may be an indicator for severity in paediatric RSV disease.

**Electronic supplementary material:**

The online version of this article (doi:10.1186/s12879-016-1454-x) contains supplementary material, which is available to authorized users.

## Background

Respiratory syncytial virus (RSV) is a major cause of severe respiratory infections in infants below 6 months of age and the most common cause for bronchiolitis. Approximately 60 % of all infants are infected with RSV during their first winter season and at the age of 2 almost all children have encountered RSV [1]. The vast majority of children will develop relatively mild symptoms, comparable to a common cold. However, approximately 2–3 % will develop bronchiolitis and will be hospitalized [2]. Known risk factors for severe disease are age (<6 months), prematurity, congenital heart or lung disease and presence of siblings [2–5]. A considerable part of the severely ill patients were previously healthy and, at this moment, we do not understand why these children become severely ill.

Mucosal surfaces of the human body are inhabited by complex microbial ecosystems, together called the ‘microbiome’. A growing body of evidence shows that the microbiome is crucial for the shaping of our immune system [6, 7]. Because an over-exuberant immune response plays a crucial role in severe RSV infections, the composition of the microbiome during the first months after birth should be considered as a potential determinant for severity of disease upon infection with RSV [8]. Interactions between RSV and *Streptococcus pneumoniae* are well-documented previously. Most of these studies focus on the influence of RSV infections on secondary pneumococcal infections, e.g. showing an enhanced adherence of *S. pneumoniae* to RSV-infected cells [9–13]. However, whether the presence of *S. pneumoniae* in the nasopharynx may influence a subsequent RSV infection has not been studied in infants. There are studies showing that *S. pneumoniae* may aggravate RSV infections [14, 15]. Cells infected by pneumococci are more susceptible to RSV infection in vitro and in a mouse model [14]. In a study in South-Africa, it was shown that vaccination against *S. pneumoniae* reduces viral-caused pneumonias by 31 %, suggesting a promoting role for *S. pneumoniae* in viral respiratory infections [15]. In this study, the presence and density of *S. pneumoniae* was determined in a clinical cohort of infants hospitalized with RSV infections. Classically, severity of an infection is thought to be dependent on two factors: pathogen load and inflammatory response. Previous studies have shown that bacterial colonization is able to influence viral infection rate [16–18], but may also influence the inflammatory response during an infection [19–22]. Therefore, we studied correlations between pneumococcal colonization patterns and RSV load, levels of the inflammatory mediators IL-6 and MMP-9, both associated with RSV infection [23–25] as well as *S. pneumoniae* infection [26–28], and severity of disease.

## Methods

### Study design

Children younger than 2 years of age with laboratory confirmed RSV infections were prospectively included during three consecutive winter seasons (November-April in 2010/2011, 2011/2012 and 2012/2013). Written informed consent was obtained from all parents. Patients with congenital heart or lung disease, immunodeficiency or glucocorticoid use were excluded. Medical history, demographics and clinical parameters were collected from questionnaires and medical records. Patients were divided into three groups. Children without hypoxia were classified as ‘mildly ill’. ‘Moderately ill’ children received supplemental oxygen, while ‘severely ill’ children required mechanical ventilation. Within 24 h after admission, a nasopharyngeal aspirate (NPA) was collected (acute) and parents from hospitalized children were asked for permission to draw a second NPA sample 4–6 weeks after admission (recovery). The study was approved by the Central Committee on Research Involving Human Subjects of the Radboud university medical center.

### Sample collection

The nasopharyngeal aspirates were collected by introducing a catheter, connected to a collection tube and an aspiration system, into the nasopharyngeal cavity. Then, 0.5 ml of saline was instilled into the catheter and, while slowly retracting the catheter, the nasopharyngeal fluid was aspirated in a collection tube. Afterwards the catheter was flushed with 1 ml of saline and this was added to the collection fluid. Samples were kept cold and were immediately transferred to the laboratory. Samples were taken for viral and bacterial diagnostics. For viral diagnostics samples were analyzed by multiplex PCR, quantifying 15 different viral pathogens, as previously described [29]. The remaining NPA was centrifuged at 500*g for 10 min at 4 °C to spin down the mucus and cells, after which the supernatant was frozen at −80 °C for ELISA.

### Bacterial diagnostics

Nasopharyngeal aspirates (300 μl) were resuspended in 343 μl lysis buffer (AGOWA mag Mini DNA Isolation Kit, AGOWA) with 57 μl protease. Then, 25–50 mg sterile zirconium beads were added and 500 μl phenol. The samples were disrupted using the TissueLyser (Qiagen) for 2 min, twice. The samples were then centrifuged for 10 min at 10,000 rpm and the supernatant containing the released DNA was then purified according to the protocol included in the AGOWA mag Mini DNA Isolation Kit, as described previously [30]. Samples were resuspended in 50 μl elution buffer and stored at −80 °C until further use. RT qPCR was used to quantify total bacterial carriage density (16 s), *S. pneumoniae* (Sp), and *H. influenzae* (Hi) by amplifying the 16 s rRNA gene, the *lytA* gene and the *hpd* gene, respectively, as previously described [30]. Primers and probes used can be found in Additional file 1: Table S1. All samples were run in duplicate. Samples were analyzed on a Bio-Rad CFX96 Real-Time System. Primer and probe concentrations were optimized for each target and the machine. Final primer/probe concentrations were 5 nM for 16 s, 200 nM for *lytA* (Sp) and 300 nM for *hpd* (Hi). 0.8 μl of each primer and/or probe was added to a 20 μl reaction volume. Standard curves were created using purified genomic DNA extracted from laboratory reference strains and quantified using the NanoDrop ND-1000. For *S. pneumoniae*, DNA was extracted from TIGR4 [31]. For *H. influenzae*, DNA was extracted from R2866 [32]. As a measure for bacterial density, we determined the number of bacterial genomes per ml of NPA. We used the following formula: Number of genome copies per μl of extracted DNA = (mass in ng * Avagadro’s number)/(genome length for each bacteria * 10^9^ * 650). 650 daltons is the average weight of a DNA basepair. We then multiplied this number by 167 to account for the difference between the volume of nasal wash used in the extraction (300 μl) and the volume of extracted DNA (50 μl) in order to determine genome copies per ml of NPA [30]. As a negative control, a water sample was included during the whole procedure from DNA isolation to RT qPCR. For the specific bacterial RT qPCR’s Ct values above 35 were regarded as negative, based on the negative controls. For the 16 s RT qPCR, Ct values above 30 were regarded as negative.

### Inflammatory markers

MMP-9 concentrations were measured in the nasopharyngeal aspirates using R&D ELISA kits (R&D systems) according to the instructions of the manufacturer. Samples were 10,000x diluted for the MMP-9 ELISA, which therefore had a lower detection limit of 156 ng/ml. IL-6 concentrations were measured using a Sanquin ELISA kit according to the instructions of the manufacturer. Samples were 100-fold diluted and therefore the IL-6 ELISA had a lower detection limit of 156 pg/ml.

### Statistical analysis

Values are expressed as percentages for categorical variables and as median and interquartile range (IQR) for continuous variables. Chi-squared tests were performed to compare categorical data between multiple groups. When significant differences were identified, Fisher’s exact tests were performed to specify which groups differed. As tested by Shapiro-Wilk’s test, none of the continuous variables were normally distributed. Therefore, Kruskal–Wallis H tests were used to compare continuous data between multiple groups. When significant differences were found, Mann–Whitney U tests were performed to specify which groups differed. To determine whether correlations existed, a Spearman correlation test was performed. A value of *p* < 0.05 was considered statistically significant. All statistical analyses were conducted in GraphPad Prism 5.03 or IBM SPSS Statistics 20.

## Results

### Patient characteristics

In total, 105 RSV-infected infants were included. From these 105 RSV infected patients, 25 patients were categorized as mildly ill meaning there was no need for oxygen support, 53 patients were moderately ill and required oxygen support, 27 patients were severely ill necessitating mechanical ventilation. Significant differences between the severity groups were found for known risk factors like age and presence of siblings, but also for hospital duration, daycare attendance, vaccination status and viral co-infections (Table 1).

**Table 1:** Patient characteristics for mild, moderate and severe infections

	Mild (*n* = 25)	Moderate (*n* = 53)	Severe (*n* = 27)	*P*-value
Age (days) (median + IQR)^a^	191 (73–538)	134 (58–323)	38 (19–55)	<0.001
Gestational age (weeks) (median + IQR)^a^	39 (37–40)	39 (37–40)	38 (37–39)	NS
Male (%)	60	52	57	NS
Hospital duration (days) (median + IQR)^a^	1 (0–5)	6 (4–9)	11 (10–14)	<0.001
Siblings (%)	60	79	89	0.037
Daycare (%)	68	50	15	0.001
Antibiotics in past 4 weeks (%)	28	8	19	NS
Vaccination according to Dutch immunisation program (%)	84	79	19	<0.001
RSV load (Ct value) (median + IQR)^a^	22 (21–27)	24 (22–30)	23 (21–27)	NS
Viral co-infection (%)	64	46	21	0.007

Data are presented as median + interquartile range (IQR) for continuous variables or in percentages for categorical variables. ^a^Shapiro–Wilk’s test was used to test data normality for continuous variables. Age, gestational age, hospital duration and RSV load were not normally distributed and therefore a Kruskal–Wallis H test was used for continuous variables. Categorical variables where tested using Chi-square analysis

### Presence of *S. pneumoniae* does not correlate with viral load, inflammation or disease severity

**Fig. 1:**
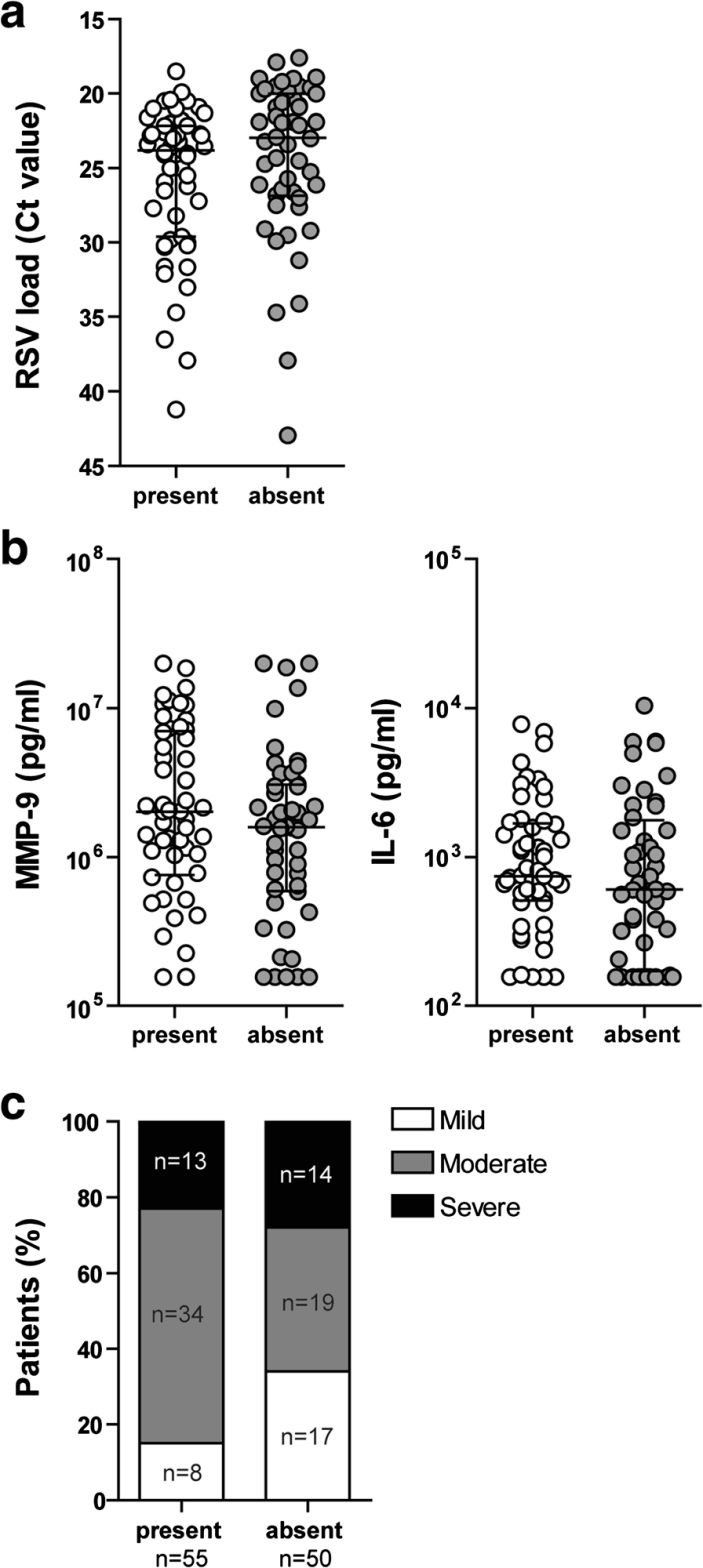
Pneumococcal presence does not correlate with RSV load, inflammation or disease severity. Viral load (**a**) and MMP-9 and IL-6 (**b**) were compared between the group positive for *S. pneumoniae* and the group negative for *S. pneumoniae*. Data shown are median ± IQR. Data were tested for significant differences using a Mann–Whitney U test. Pneumococcal presence was compared between the three severity groups (**c**). Differences in bacterial colonization rates were compared using Chi-square tests. When significant differences were found, Fisher’s exact tests were performed to specify which groups differed significantly. The moderately ill infants had a significantly higher colonization rate compared to the mildly infected infants (***p < 0.001)

In all collected samples, 16 s rRNA could be detected by RT qPCR. Samples could therefore be used for additional analysis (data not shown). First, we studied whether pneumococcal colonization influenced RSV infection. There were no significant differences between the pneumococcal positive group compared to the pneumococcal negative group, regarding viral load (Fig. 1a). This was also the case for the inflammatory mediators, MMP-9 and IL-6 (Fig. 1b). When looking at severity scores, the moderately ill infants had a higher percentage of pneumococcal positive infants compared to the mildly ill infants (Fig. 1c). However, we did not see this trend for the severe group.

In conclusion, we detected no correlation between the presence of *S. pneumoniae* and viral load, inflammation or severity of disease. As shown in Table 1, age is an important potential confounder. Therefore, we checked whether pneumococcal colonization was correlated with age (Additional file 1: Figure S1A). Some significant differences were found between the different age groups but no clear trends were found. The same analyses were performed for another potentially pathogenic nasopharyngeal bacterium (*Haemophilus influenzae*) to see if the effects found were specific for *S. pneumoniae*. No significant differences regarding viral load, inflammation and severity were found for *H. influenzae* (Additional file 1: Figure S2). Colonization with *H. influenzae* was also not age-dependent (Additional file 1: Figure S1B).

### Pneumoccocal density correlates with viral load, inflammation and severity

**Fig. 2:**
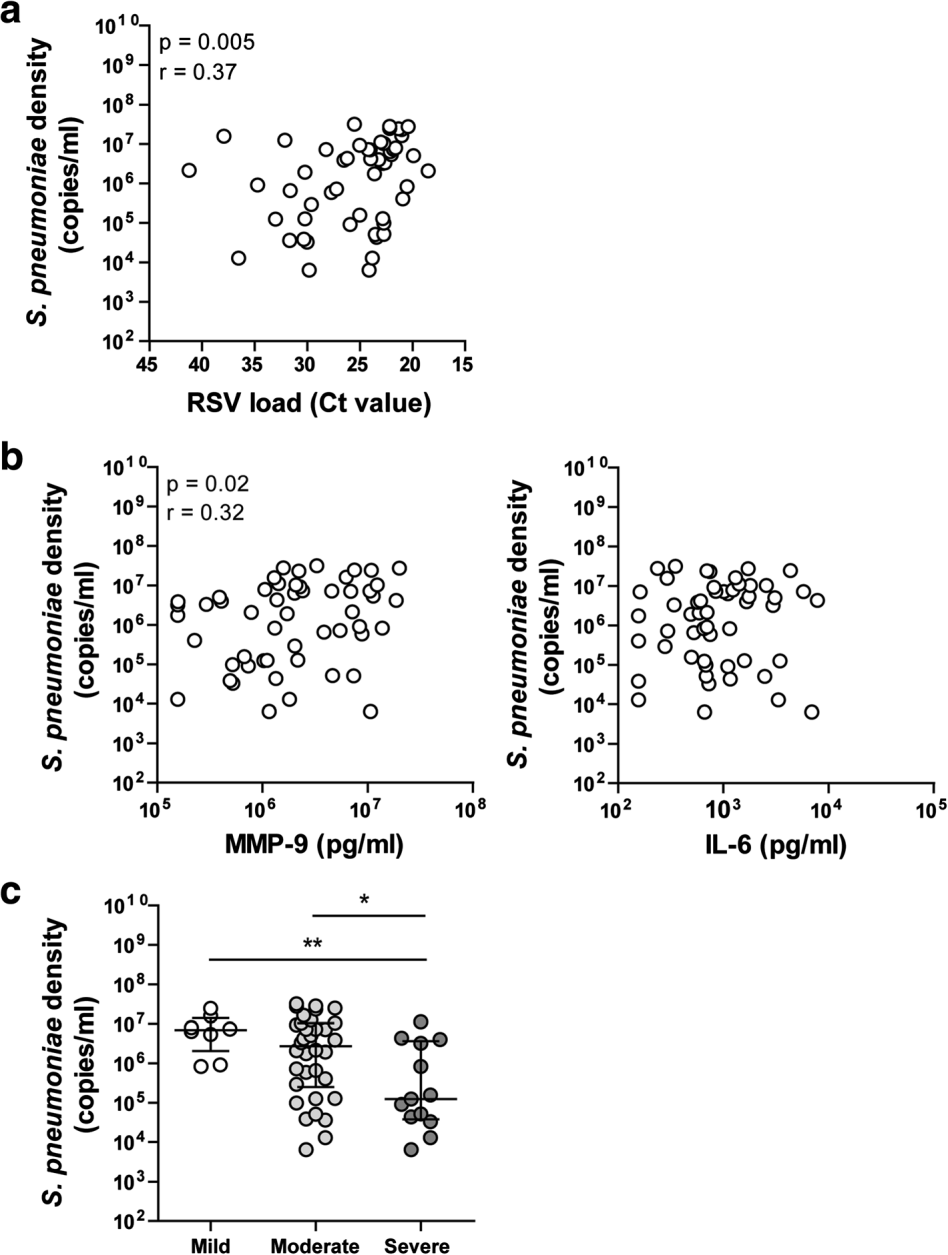
Pneumococcal density correlates with RSV load, inflammation and severity. Pneumococcal density was correlated with viral load (**a**) and MMP-9 and IL-6 levels (**b**). Correlations were tested for significance using a Spearman correlation test. Pneumococcal density was compared between the three severity groups (**c**). Data shown are median ± IQR. Differences in bacterial carriage density are tested using a Kruskal-Wallis test. When significant differences were found, Mann–Whitney U tests were performed to specify which groups differed (*p < 0.05, **p < 0.01)

As we did not see any differences between patients with or without pneumococcus, we then focussed on the patients that were pneumococci positive. We studied whether pneumococcal density influences RSV infection. When correlating pneumococcal density with RSV load, we found that a higher density of *S. pneumoniae* was correlated with higher titers of RSV (Fig. 2a). This also holds true for MMP-9, for which a higher *S. pneumoniae* density correlated with higher concentrations of MMP-9 (Fig. 2b). In contrast, IL-6 did not correlate with pneumococcal density (Fig. 2b). Lastly, we also compared the different severity groups and found that more severely infected infants had lower pneumococcal densities (Fig. 2c). In addition, pneumococcal density was not significantly different between the different age groups (Additional file 1: Figure S3A) and therefore not age-dependent.

*H. influenzae* was also included, but no significant differences were found (Additional file 1: Figure S4) and *H. influenzae* density was not age-dependent (Additional file 1: Figure S3B). These results indicate that the effects found are specific for *S. pneumoniae*.

### Pneumococcal colonization and density does not change after RSV infection

**Fig. 3:**
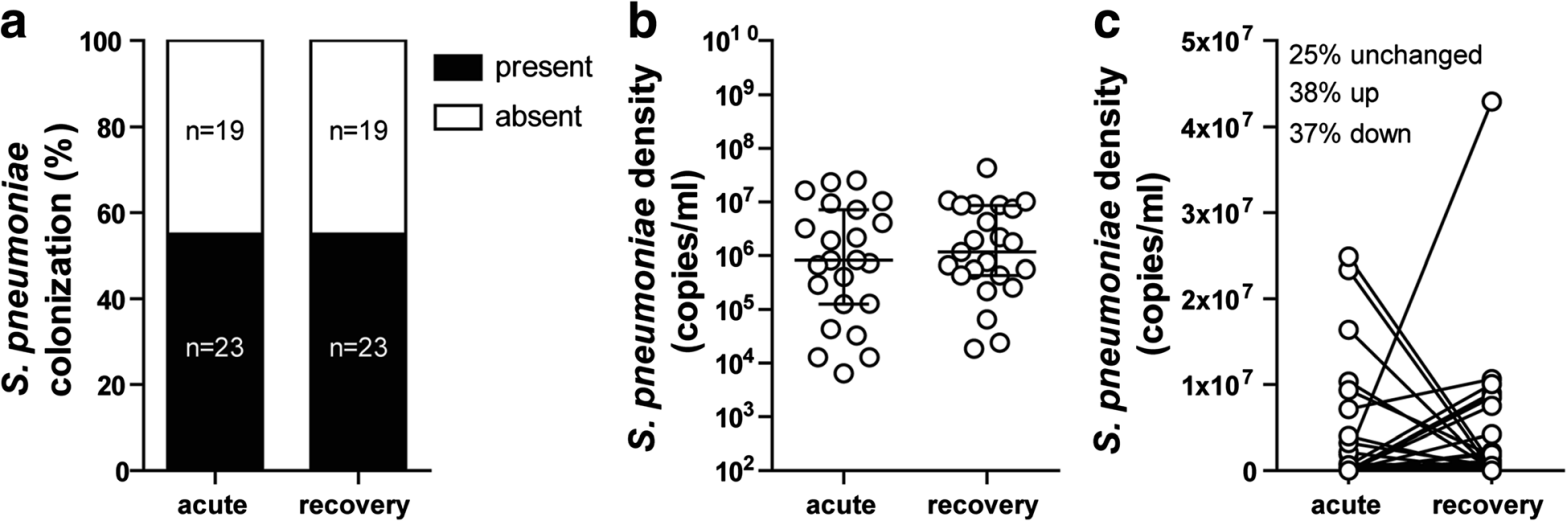
Pneumococcal colonization and density does not change after RSV infection. Pneumococcal colonization rate, density and shifts were compared between the acute and the recovery group. Differences in bacterial colonization rates were compared using a Fisher’s exact test of all patients that had an acute and recovery sample taken (**a**). Differences in bacterial density between de pneumococcal positive samples were compared using a Mann–Whitney U test. Data shown are median ± IQR (**b**). Shifts in pneumococcal density between all acute and recovery samples are shown (**c**)

Additionally, 42 of the patients were also sampled 4–6 weeks after hospital discharge. Because viral respiratory infections are known to increase the risk of bacterial infections, we investigated whether pneumococcal colonization rates and density change after an RSV infection. Percentages of colonization were identical when comparing the acute and recovery group (Fig. 3a). Therefore, RSV infection did not change colonization rates in infants. Also, pneumococcal density of the pneumococcal positive patients was not significantly different between patients having an infection and patients in the recovery phase (Fig. 3b). Lastly, when we looked at the shifts in colonization, we saw that 25 % of the infants was not colonized during the acute infection and remained so during the recovery phase (Fig. 3c). Also, approximately the same number of infants acquired *S. pneumoniae* after an RSV infection as the number losing *S. pneumoniae*.

## Discussion

This study is the first to report that *S. pneumoniae* density in the nasopharynx is correlated with viral load, inflammatory conditions and severity during RSV infection of hospitalized infants. We here show that pneumococcal carriage density is lower during severe RSV infections and that a low density correlates with a low RSV load and low levels of MMP-9. This indicates that *S. pneumoniae* colonization during RSV infection is associated with viral infection and the inflammatory response and therefore could influence the development of disease.

We used RT qPCR to determine bacterial carriage density instead of culturing methods. RT qPCR is faster and more sensitive than culturing techniques, especially when samples have to be frozen [33, 34]. Good correlations have been reported for *S. pneumoniae* and *H. influenzae* when comparing RT qPCR with quantitative culturing [35]. One important drawback of RT qPCR is that viable and nonviable bacteria cannot be distinguished. However, for our study, this is an advantage as it more accurately detects the presence of bacterial pathogens without any disturbance due to recent antibiotic treatment. In the Netherlands, patients with severe infections, who require mechanical ventilation, receive selective decontamination of the digestive tract (SDD) by use of antibiotics. Our study subjects were sampled within 24 h after admission to the hospital. Therefore, we do not expect that the SDD regimen will have exerted an effect on bacterial carriage density measured by RT qPCR. Moreover, if SDD antibiotics would have such rapid effects, one would also expect to see the same trends for *H. influenzae*, but this is not the case.

A recent study has shown that antibiotics given in the week prior to sampling reduced colonization rates of potential pathogenic bacteria, e.g. *S. pneumoniae*, *M. catarrhalis*, *H. influenzae*, *S. aureus* and *β-hemolytic Streptococcus* but had no clear effect on paediatric RSV disease severity scores [36]. In our study, we determined whether infants received antibiotic treatment in the 4 weeks prior to hospitalization. No significant differences were found in pneumococcal colonization or severity status (data not shown).

We included a control group of infants who were hospitalized for a hernia operation to check how the inflammatory mediators behave in different groups. We see different dynamics for MMP-9 and IL-6 (Additional file 1: Figure S5). IL-6 is only elevated during the acute phase of disease, whereas MMP-9 is elevated both during the acute phase of disease and the recovery phase.

To our knowledge, we are the first to show a negative correlation between RSV disease severity and pneumococcal carriage density. Clinical studies have shown that the pneumococcal conjugate vaccine also results in a reduction of 31 % of the viral respiratory tract infections [15, 37]. This suggests that colonization with *S. pneumoniae* increases the risk of viral infection or that its presence enhances symptoms. However, our study shows that severely ill patients have lower pneumococcal carriage density, thus suggesting that high *S. pneumoniae* density protects against severe infections.

*S. pneumoniae* and RSV are known to influence each other [38]. In vitro studies have shown that RSV infection of respiratory epithelial cells enhances the adherence of *S. pneumoniae*, possibly because the RSV G protein can serve as a receptor for *S. pneumoniae* [9, 12]. This is in accordance with our observation that higher RSV loads coincide with higher pneumococcal carriage density.

MMP-9 is a matrix metalloproteinase which is involved in breakdown of extracellular matrix and appears to be a regulatory factor in neutrophil migration across the basement membrane [39]. High MMP-9 concentrations have been associated with severe RSV infections [24]. Studies have shown that RSV is able to induce MMP-9 production [40]. However, in our study no correlation was found between MMP-9 levels and RSV load (data not shown). Previously, we have shown that *S. pneumoniae* is able to induce high MMP-9 levels under in vitro conditions [26]. This is supported by this study where we show a correlation between pneumococcal carriage density and MMP-9 levels, in which higher numbers of *S. pneumoniae* led to higher concentrations of MMP-9.

All our data together suggest that severely ill infants have lower pneumococcal loads, these lower pneumococcal loads are correlated with lower RSV levels and lower MMP-9 levels. However, this suggests indirectly that severely ill infants have lower RSV levels, which seems contradictory. Although the general consensus is that viral load is probably correlated with disease severity, there is still discussion to what extent [41–44]. Our data do not show a correlation between RSV load and disease severity (Table 1). A possible explanation for this discrepancy in our results is that at the moment the infants are included, they are in an advanced stage of disease. It is therefore possible that in the severe cases the virus has already partly been cleared and that severe inflammation is cause of the severity, not viral load. Another explanation is that the interactions between *S. pneumoniae*, RSV load, inflammation and disease severity are multidirectional and more complex than we can grasp in this study. Other factors, that we did not include in this study, could play a role. We did carefully evaluated the presence of potential confounders that may explain the results of our study. When comparing severity groups, the severe groups had a lower daycare attendance and lower vaccination rates. These are both due to the fact that the severely ill patients were often too young for vaccination and daycare attendance. The severely infected patients also had more siblings, which is a known risk factor for severe RSV infections [4, 45]. Vaccination, daycare attendance and presence of siblings can all influence pneumococcal carriage patterns. However, the severely infected infants did not yet receive pneumococcal vaccination and had more siblings. This would all have resulted in a higher pneumococcal load instead of a lower load. We cannot exclude that less daycare attendance in the severely infected infants may have contributed to lower pneumococcal carriage. Patients with severe infections had more RSV mono-infections compared to the other severity groups. This was already shown by our group in a previous cohort [3]. Finally, age could be a confounder as the severely ill patients are significantly younger compared to the moderate or mildly ill patients. As we have shown in Additional file 1: Figure S1 and S3, *S. pneumoniae* and *H. influenzae* carriage densities were not dependent on age, whereas the densities of *S. aureus* and *M. catarrhalis* were age dependent (data not shown).

There are some limitations to our study. Based on the study design we cannot state anything on causality. We do not know whether pneumococcal density changes as a result of RSV infection, or whether the pneumococcal density was already different and potentially influenced susceptibility to an RSV infection. A prospective study is needed to definitely determine whether *S. pneumoniae* influences RSV severity. Moreover, there is a difference between absolute density and relative abundance. We did not look at the influence of relative abundance of *S. pneumoniae*.

## Conclusions

In summary, we here show that *S. pneumoniae* density correlates to disease severity, viral load and inflammatory mediators, which suggests that *S. pneumoniae* density influences both viral load as well as the mucosal inflammatory response during an RSV infection. Once we understand the role of different bacteria residing in the upper respiratory tract in severity of RSV infections, we might be able to predict severity or modify the composition of the microbiome to prevent severe infections.

## Additional file

Additional file 1: Table S1. Primers and probes used for RT qPCR assays. **Figure S1.** No clear age dependent colonization patterns were found for S. pneumoniae (A) and H. influenza (B). **Figure S2.** H. influenzae presence does not influence RSV load, inflammation or severity. **Figure S3.** S. pneumoniae (A) and H. influenza (B) density are not age dependent. **Figure S4.** H. influenzae density does not influence RSV load, inflammation and severity. **Figure S5.** IL-6 levels are elevated during acute phase of disease, whereas MMP-9 is elevated during acute and recovery phase of disease. (PDF 2 MB)
