# Supplementary material for: High pneumococcal density correlates with more mucosal inflammation and reduced respiratory syncytial virus disease severity in infants
Source: BMC Infect Dis. 2016 Mar 17;16:129. doi: 10.1186/s12879-016-1454-x (PMC4794819; doi:10.1186/s12879-016-1454-x)
Supplement: Additional file 1: Table S1. — Primers and probes used for RT qPCR assays. Figure S1. No clear age dependent colonization patterns were found for S. pneumoniae (A) and H. influenza (B). Figure S2. H. influenzae presence does not influence RSV load, inflammation or severity. Figure S3. S. pneumoniae (A) and H. influenza (B) density are not age dependent. Figure S4. H. influenzae density does not influence RSV load, inflammation and severity. Figure S5. IL-6 levels are elevated during acute phase of disease, whereas MMP-9 is elevated during acute and recovery phase of disease. (PDF 2 MB) [file 12879_2016_1454_MOESM1_ESM.pdf]

Additional file 1

Additional file 1: Table S1. Primers and probes used for RT qPCR assays

Species (gene)	Primer/probe	5'-3' nucleotide sequence	Source
**Bacterial carriage density (16s)**	forward primer	CGAAAGCGTGGGGAGCAAA	[1]
	reverse primer	GTTCGTACTCCCCAGGCGG	
S. pneumoniae **(lyta)**	forward primer	ACGCAATCTAGCAGATGAAGCA	[2]
	reverse primer	TCGTGCGTTTTAATTCCAGCT	
	probe	GCCGAAAACGCTTGATACAGGGAG *	
H. influenzae **(hpd)**	forward primer	AGATTGGAAAGAAACACAAGAAAAAGA	[3]
	reverse primer	CACCATCGGCATATTTAACCACT	

***The probe was labelled with FAM at 5'-end and Black Hole Quencher (BHQ) at the 3'-end.**

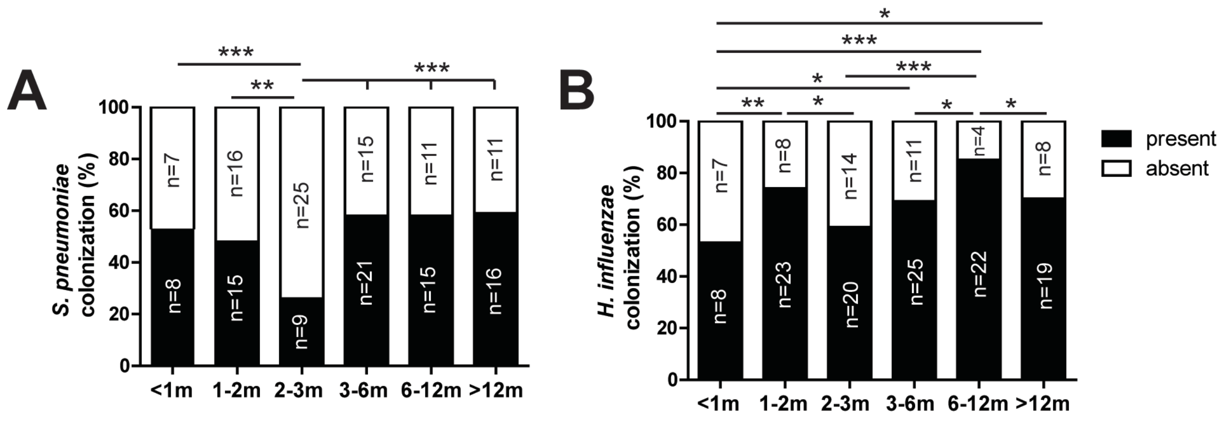

Additional file 1: Figure S1. No clear age dependent colonization patterns were found for *S. pneumoniae* (A) and *H. influenza* (B). Differences in bacterial colonization rates were compared using Chisquare tests. When significant differences were found, Fisher's exact tests were performed to specify which groups differed. Significant differences were found but no clear trend was visible.

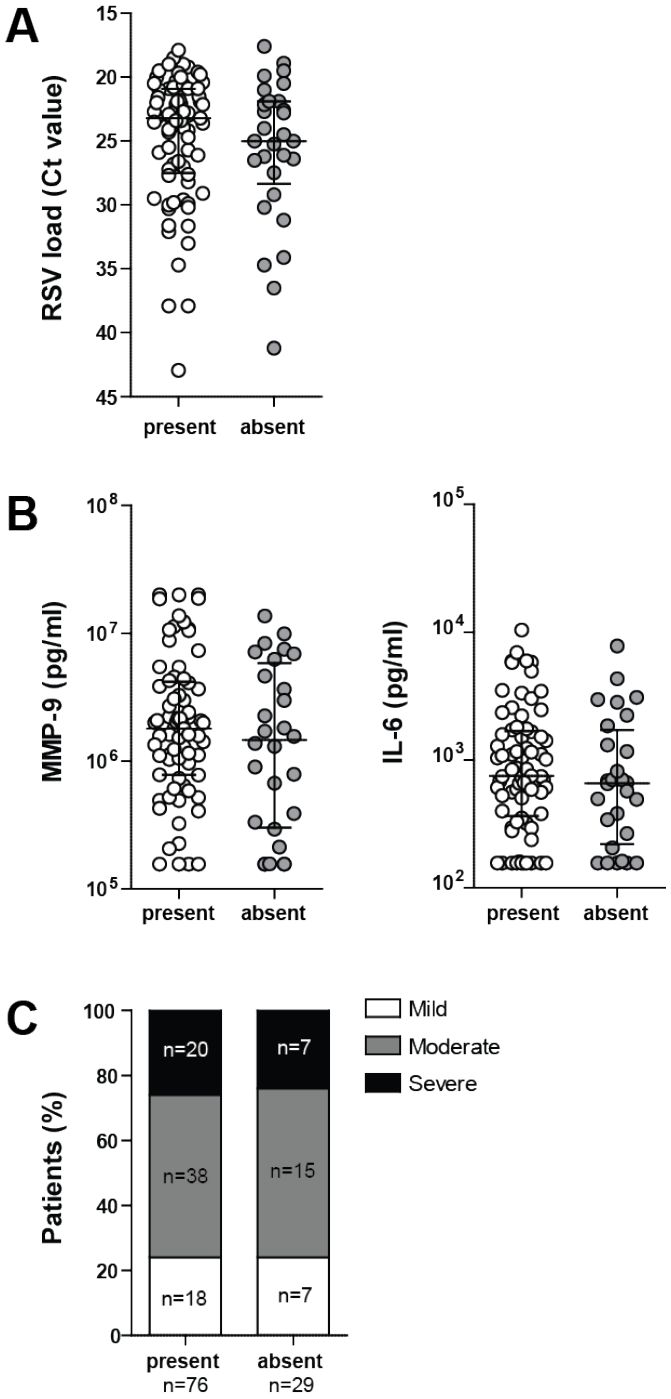

Additional file 1: Figure S2. ***H. influenzae*** **presence does not influence RSV load, inflammation or severity.** Viral load (**A**) and MMP-9 and IL-6 (**B**) were compared between the group positive for *H. influenzae* and the group negative for *H. influenzae*. Data shown are median ± IQR. Data were tested for significant differences using a Mann-Whitney U test. *H. influenzae* presence was compared between the three severity groups (**C**). Differences in bacterial colonization rates were compared using Chi-square tests. No significant differences were found.

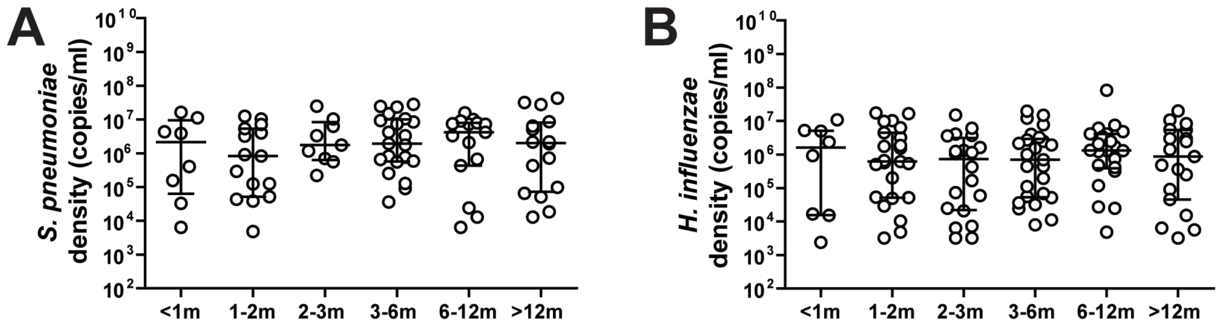

Additional file 1: Figure S3. ***S. pneumoniae*** **(A) and** ***H. influenza*** **(B) density are not age dependent.** Data shown are median ± IQR. Differences in bacterial densities are tested using the Kruskal-Wallis test. No significant differences were found.

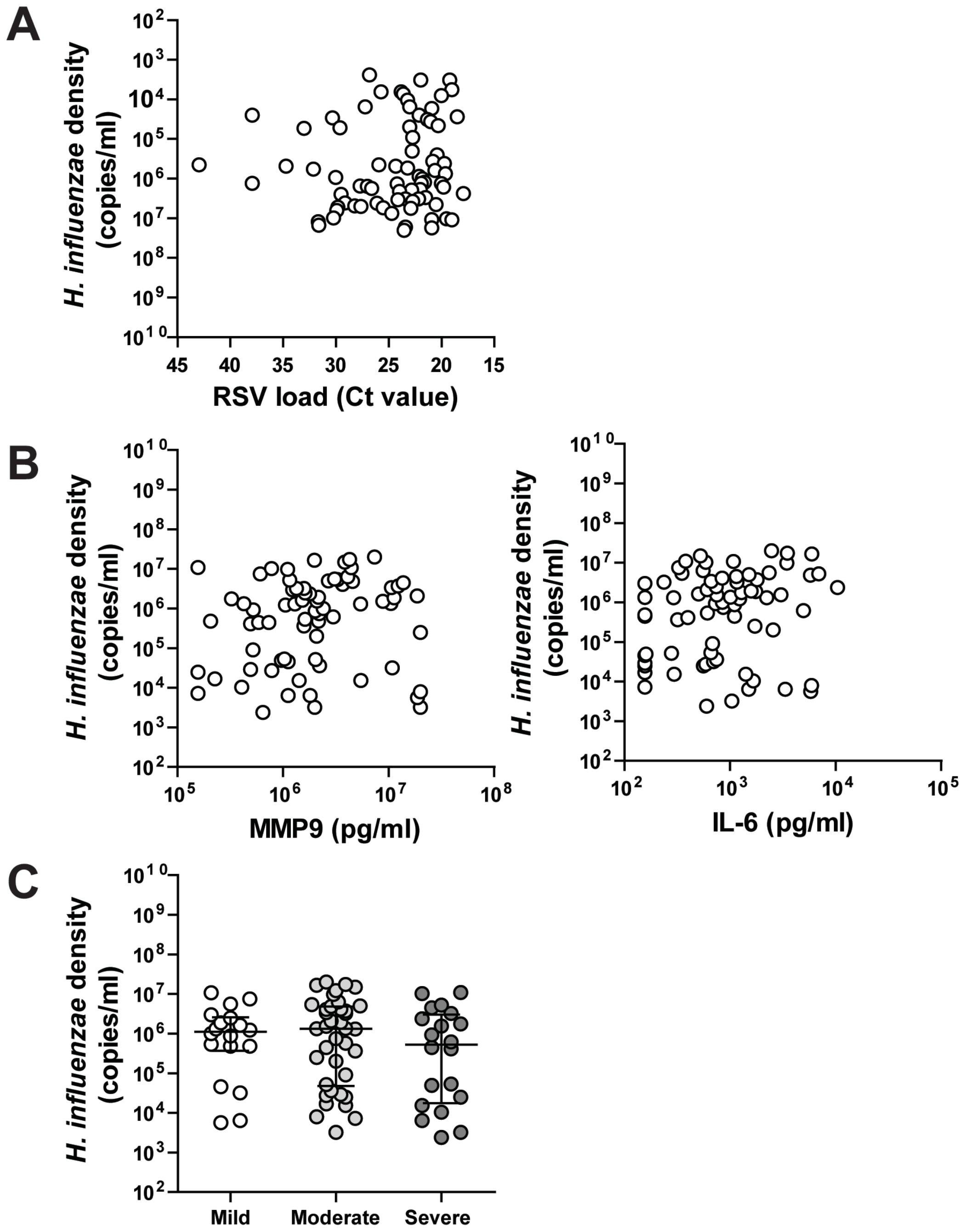

Additional file 1: Figure S4. ***H. influenzae*** **density does not influence RSV load, inflammation and severity.** *H. influenzae* density was correlated with viral load (**A**) and MMP-9 and IL-6 levels (**B**). Correlations were tested for significance using a Spearman correlation test. *H. influenzae* carriage density was compared between the three severity groups (**C**). Data shown are median ± IQR. Differences in bacterial carriage density are tested using the Kruskal-Wallis test. No significant differences were found.

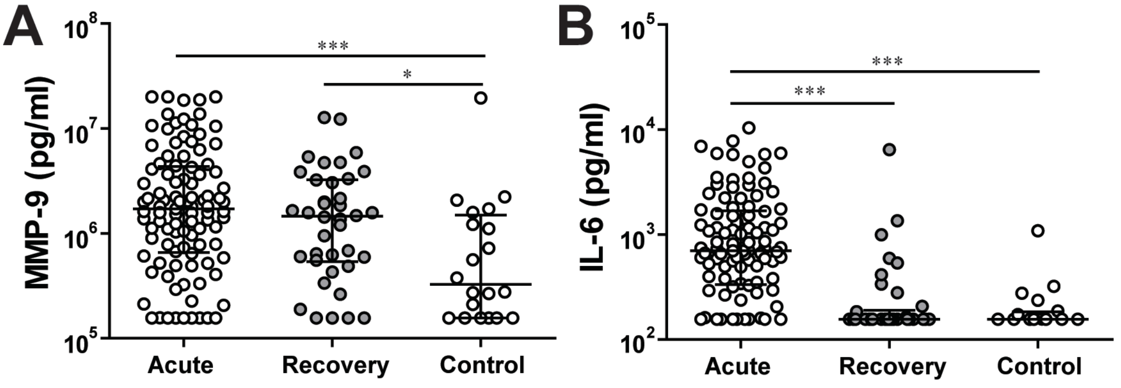

Additional file 1: Figure S5. IL-6 levels are elevated during acute phase of disease, whereas MMP-9 is elevated during acute and recovery phase of disease. MMP-9 levels **(A)** and IL-6 levels **(B)** were measured during the acute and recovery phase of disease and were compared to a control group of healthy patients. Data shown are median ± IQR. Differences in cytokine levels are tested using the Kruskal-Wallis test. When significant differences were found the different groups were compared using a Mann-Whitney U test (*p<0.05, ***p<0.001).

References

1. Bogaert D, Keijser B, Huse S, Rossen J, Veenhoven R, Van Gils E, Bruin J, Montijn R, Bonten M, Sanders E: **Variability and diversity of nasopharyngeal microbiota in children: a metagenomic analysis**. *PloS one* 2011, **6**(2):e17035.
2. Maria da Gloria SC, Tondella ML, McCaustland K, Weidlich L, McGee L, Mayer LW, Steigerwalt A, Whaley M, Facklam RR, Fields B: **Evaluation and improvement of real-time PCR assays targeting lytA, ply, and psaA genes for detection of pneumococcal DNA**. *Journal of clinical microbiology* 2007, **45**(8):2460-2466.
3. Wang X, Mair R, Hatcher C, Theodore MJ, Edmond K, Wu HM, Harcourt BH, Maria da Gloria SC, Pimenta F, Nymadawa P: **Detection of bacterial pathogens in Mongolia meningitis surveillance with a new real-time PCR assay to detect Haemophilus influenzae**. *International Journal of Medical Microbiology* 2011, **301**(4):303-309.
